# Cyclooxygenase-2 Inhibition as an Add-On Strategy in Drug Resistant Epilepsy—A Canine Translational Study

**DOI:** 10.3389/fvets.2022.864293

**Published:** 2022-04-07

**Authors:** Andrea Fischer, Velia-Isabel Hülsmeyer, Viviana P. Munoz Schmieder, Andrea Tipold, Marion Kornberg, Florian König, Felix K. Gesell, Liza K. Ahrend, Holger A. Volk, Heidrun Potschka

**Affiliations:** ^1^Clinic of Small Animal Medicine, Centre for Clinical Veterinary Medicine, Ludwig-Maximilians-University Munich (LMU), Munich, Germany; ^2^Department of Small Animal Medicine and Surgery, University of Veterinary Medicine, Hannover, Germany; ^3^AniCura Tierklinik Trier, Trier, Germany; ^4^Small Animal Practice Dr. Florian König, Wiesbaden, Germany; ^5^Institute of Pharmacology, Toxicology, and Pharmacy, Ludwig-Maximilians-University Munich, Munich, Germany

**Keywords:** idiopathic epilepsy, coxib, P-glycoprotein, seizures, dog, pharmacoresistance, treatment study, blood brain barrier

## Abstract

Drug-resistant epilepsy is a common complaint in dogs and affects up to 30% of dogs with idiopathic epilepsy. Experimental data suggest that targeting cyclooxygenase-2 (COX-2) mediated signaling might limit excessive excitability and prevent ictogenesis. Moreover, the role of COX-2 signaling in the seizure-associated induction of P-glycoprotein has been described. Thus, targeting this pathway may improve seizure control based on disease-modifying effects as well as enhancement of brain access and efficacy of the co-administered antiseizure medication. The present open-label non-controlled pilot study investigated the efficacy and tolerability of a COX-2 inhibitor (firocoxib) add-on therapy in a translational natural occurring chronic epilepsy animal model (client-owned dogs with phenobarbital-resistant idiopathic epilepsy). The study cohort was characterized by frequent tonic–clonic seizures and cluster seizures despite adequate phenobarbital treatment. Enrolled dogs (*n* = 17) received a firocoxib add-on therapy for 6 months. Tonic–clonic seizure and cluster seizure frequencies were analyzed at baseline (6 months) months during the study (6 months). The responders were defined by a substantial reduction of tonic–clonic seizure and cluster seizure frequency (≥50%). In total, eleven dogs completed the study and were considered for the statistical analysis. Two dogs (18%, 2/11) were classified as responders based on their change in seizure frequency. Interestingly, those two dogs had the highest baseline seizure frequency. The overall tolerability was good. However, given the low percentage of responders, the present data do not support an overall considerable efficacy of COX-2 inhibitor add-on therapy to overcome naturally occurring phenobarbital-resistant epilepsy in dogs. Further translational evaluation should only be considered in the canine patients with a very high baseline seizure density.

## Introduction

Inflammation is considered as a major contributor to ictogenesis in the chronic epileptic brain (1). Controversial data have been obtained from the rodent experiments indicating beneficial, detrimental, or lack of effects of strategies targeting the inflammatory enzyme cyclooxygenase-2 (COX-2) (1). Studies supporting the targeting of COX-2 reported delays in seizure onset, reduced seizure duration, and increased seizure thresholds (2–4). Moreover, data from a chronic rat model of drug-resistant temporal lobe epilepsy suggested that COX-2 inhibition might even help to overcome drug resistance (5). According to the previous findings, this might be related to the prevention of a seizure-induced P-glycoprotein (Pgp) upregulation and associated increases in blood–brain barrier efflux (6–8). As emphasized in a recent review, no clinical studies have yet been conducted with COX-2 selective inhibitors (=Coxibs) in human patients with epilepsy due to the withdrawal of these drugs (1). In this context, the authors pointed out that there is a particular interest to further explore the potential therapeutic benefits although the evidence is still inconclusive. Dogs with idiopathic epilepsy may be particularly suited for further investigation of this treatment strategy, because several Coxibs are currently licensed for therapeutic management of inflammatory disorders in dogs, and difficult-to-treat epilepsy with tonic–clonic and cluster seizures is a common complaint in dogs (9).

Seizure freedom is only infrequently achieved despite lifelong treatment with antiseizure drugs marketed for dogs and humans (9–12). In this context it is important to note that indicators of neuroinflammation were present in the serum of dogs with the idiopathic epilepsy (13, 14). Analysis of post mortem tissue from canine patients demonstrated upregulation of endothelial Pgp in the brains of epileptic dogs following status epilepticus or cluster seizures (15). Moreover, a high-seizure density has been identified as a risk factor for drug-resistant epilepsy in client-owned dogs (9, 11, 12). Thus, canine patients with phenobarbital-resistant epilepsy might serve as a valuable translational model allowing the assessment of COX-2 selective inhibitors. Furthermore, evaluation in dogs with spontaneous epilepsy would allow further evaluation of this treatment strategy under the clinical conditions with a higher variance in the genetic background, etiology, and seizure history as compared with highly standardized experimental rodent studies (16).

We hypothesized that COX-2 inhibition can improve seizure control in dogs with the chronic epilepsy. Therefore, we obtained pilot data on the efficacy and tolerability of a COX-2 inhibitor add-on treatment in a canine cohort with phenobarbital-resistant idiopathic epilepsy and frequent tonic–clonic seizures.

## Materials and Methods

**Figure 1:**
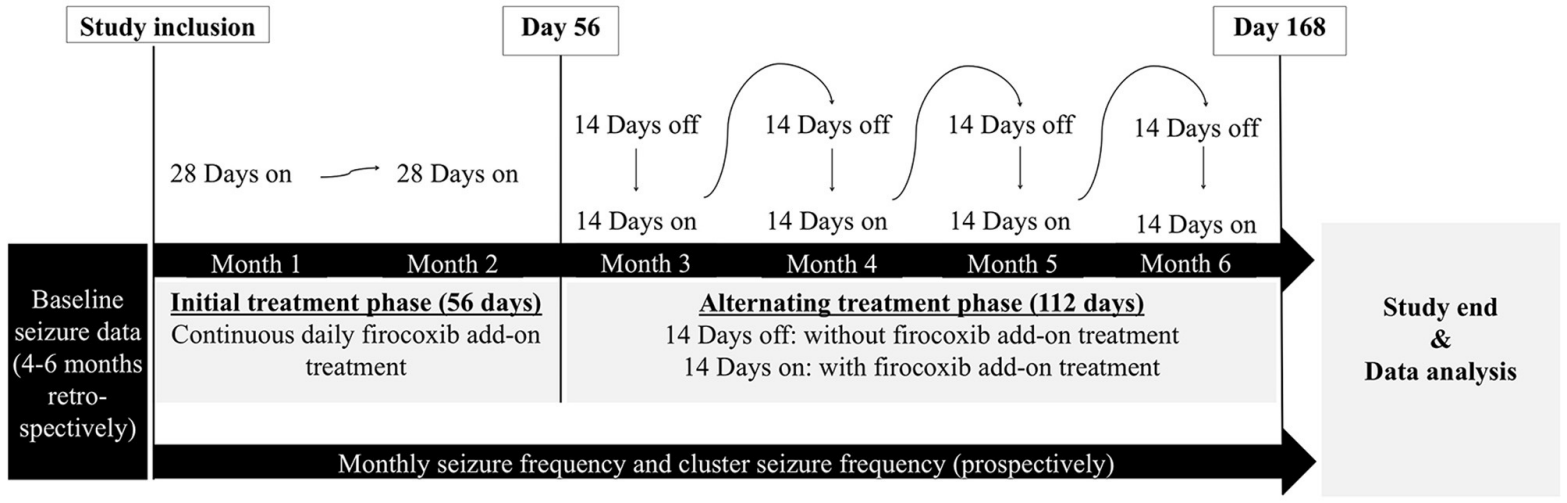
Schematic illustration of the study design. The study course consisted of a continuous treatment period for the first 56 days, followed by a 14-day alternating treatment period for 112 days (total study period 168 days). The alternating treatment protocol was selected to lower the risk of potential firocoxib adverse events. Antiseizure medication was not changed during the firocoxib add-on phase, use of diazepam rectal tubes for emergency management of seizures was allowed. Clinical examinations and blood tests were performed on a regular base throughout the entire study period. Study participation was denied to any dog with pre-existing signs of gastrointestinal disease, renal disease or pre-treatment with any non-steroidal anti-inflammatory drug or glucocorticoids.

The present clinical trial was conducted as a non-controlled open-label prospective pilot study. The ethical approval of the project was granted by the ethics and welfare committee (URN 2012 1175). All the experiments were conducted in accordance with the German animal protection law (Tierschutzgesetz). All the clients signed permission for scientific use of the data following informed consent. Client-owned pet dogs diagnosed with phenobarbital-resistant idiopathic epilepsy and frequent tonic–clonic seizures were recruited. Idiopathic epilepsy was diagnosed if recurrent seizures occurred for at least 6 months, onset was between 6 months and 6 years and clinical examination, interictal neurological examination, laboratory investigation, and MRI or CT of the brain revealed no the relevant abnormalities. A consistent high seizure frequency (≥2 tonic–clonic seizures/month in the 4 months before the study inclusion despite adequate phenobarbital therapy for at least 6 months (target steady state serum concentration 20–35 μg/ml) was required, regardless of type or dosage of other co-administered antiseizure medications. Exclusion criteria were age of onset ≥6 years, treatment with glucocorticoids in the previous 4 weeks, a history of gastrointestinal disease in the previous 6 month, a history of hepatic, renal, or cardiac disease or bleeding problems, poor documentation of the epileptic seizures and/or poor owner compliance. Owners were provided with seizure logs for documentation on first contact. Firocoxib^1^, a selective COX-2 inhibitor licensed for the treatment of osteoarthritis and post-operative pain in dogs was used as study drug. Firocoxib was administered as add-on to antiseizure medications with an oral dose of 5 mg/kg SID. Dog owners were blinded as to the exact nature of the drug. The study consisted of a continuous and an alternating treatment phase (detailed study design is illustrated in Figure 1). Seizure data were analyzed at baseline (at least 4 months, when available 6 months and during firocoxib add-on treatment (at least 4 months, when available 6 months, prospectively) by screening of seizure logs on each control visit, at 1, 2, 4, and 6 months. Seizure frequency (mean number of tonic-clonic seizures/month; in the case of cluster seizures each seizure was counted as one event), seizure days (mean number of seizure days/month), and cluster seizures (mean number of cluster days/month; each day with ≥2 seizures was counted as one event) were assessed. Phenobarbital serum concentrations (Epilepsy centre Bethel, Bielefeld, Germany), blood tests (hematology, serum chemistry, and electrolytes), urinalysis (specific gravity, pH, protein, glucose, hemoglobin, microscopic examination, and urine protein creatinine ratio) and buccal mucosal bleeding time were measured at baseline and encouraged on each control visit. Bile acid stimulation test was only performed at baseline and study exit. Primary outcome was decrease in mean seizure frequency compared with the baseline. The responders were defined by a seizure frequency reduction of ≥50% compared with the baseline data (partial treatment success). Secondary outcome was a decrease in cluster seizures compared to baseline.

Dogs with a study participation of at least 4 months were considered for statistical analysis. Statistical analysis (GraphPad Prism version 5) was performed by the Wilcoxon matched-pairs signed rank test (two-tailed) for seizure data. Statistical significance was defined at *p* ≤ 0.05.

## Results

In total, seventeen dogs (7 females; 10 males; mean age 5.12 years, range 2.0 to 11.0 years) with phenobarbital-resistant idiopathic epilepsy were recruited. Ten dogs were treated with phenobarbital monotherapy, and seven dogs with combination therapy: phenobarbital/potassium bromide (4 dogs), phenobarbital/levetiracetam (*n* = 2) or phenobarbital/potassium bromide/levetiracetam (1 dog). In seven dogs, the study was terminated early (range 8 days−16 weeks). Reasons for early study exit were severe disease of the owner (1 dog), other diseases requiring additional medication (4 dogs; vomiting and diarrhea with suspicion of pancreatitis; diarrhea; atopic dermatitis; cranial cruciate ligament rupture) and worsening of seizure control (2 dogs, day 86, day 125). One of the drop-out patients completed the minimum required study period of 4 months and was therefore included in the statistical analysis.

In total, seizure data of eleven dogs with phenobarbital-resistant idiopathic epilepsy (7 males, 4 females; mean age 5.0 years; mean body weight 21.3 kg) were analyzed. The mean age of onset was 2.9 years (range 1.0–5.0 years), duration of the epilepsy 2.1 years (range 0.5–5.0 years), and the mean phenobarbital serum concentration 29.05 μg/ml (range 24.6–32.4 μg/ml; 10 dogs). One dog with a phenobarbital serum concentration of 18 μg/ml despite high daily dosages of phenobarbital (5.6 mg/kg q12h) was included (#9). Five of these dogs received additional antiseizure medications (#12, #15, #17 potassium bromide; #9 levetiracetam; #4 potassium bromide, and levetiracetam). Three other dogs were previously treated with potassium bromide but treatment was discontinued due to lack of efficacy (#3, #8) and side effects (#8).

**Figure 2:**
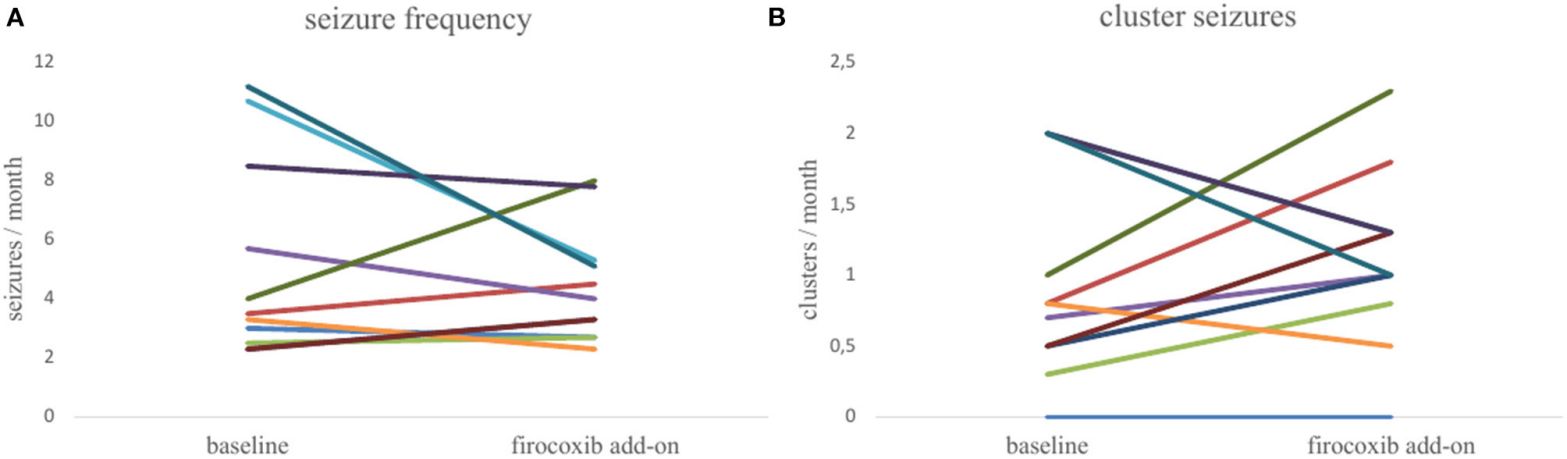
Pairwise comparisons of the monthly seizure and cluster seizure frequency of the study population (*n* = 11) before and during firocoxib add-on treatment. **(A)** Seizure frequency: The median seizure frequency for the study population at baseline and during firocoxib add-on treatment was 3.5 (range 2–11) and 4.0 (range 2–8), respectively. The median seizure frequency did not significantly differ between the baseline period and the firocoxib add-on treatment phase (*p* =0.59). Seizure frequency decreased considerably (≥50%) in two dogs with the highest seizure frequency. **(B)** Cluster seizure frequency: The median cluster seizure frequency at baseline and during firocoxib add-on treatment was 0.8 (range 0–2) and 1.0 (range 0–2), respectively. The cluster seizure frequency did not significantly differ between the baseline period and the firocoxib add-on treatment phase (*p* = 0.57).

**Figure 3:**
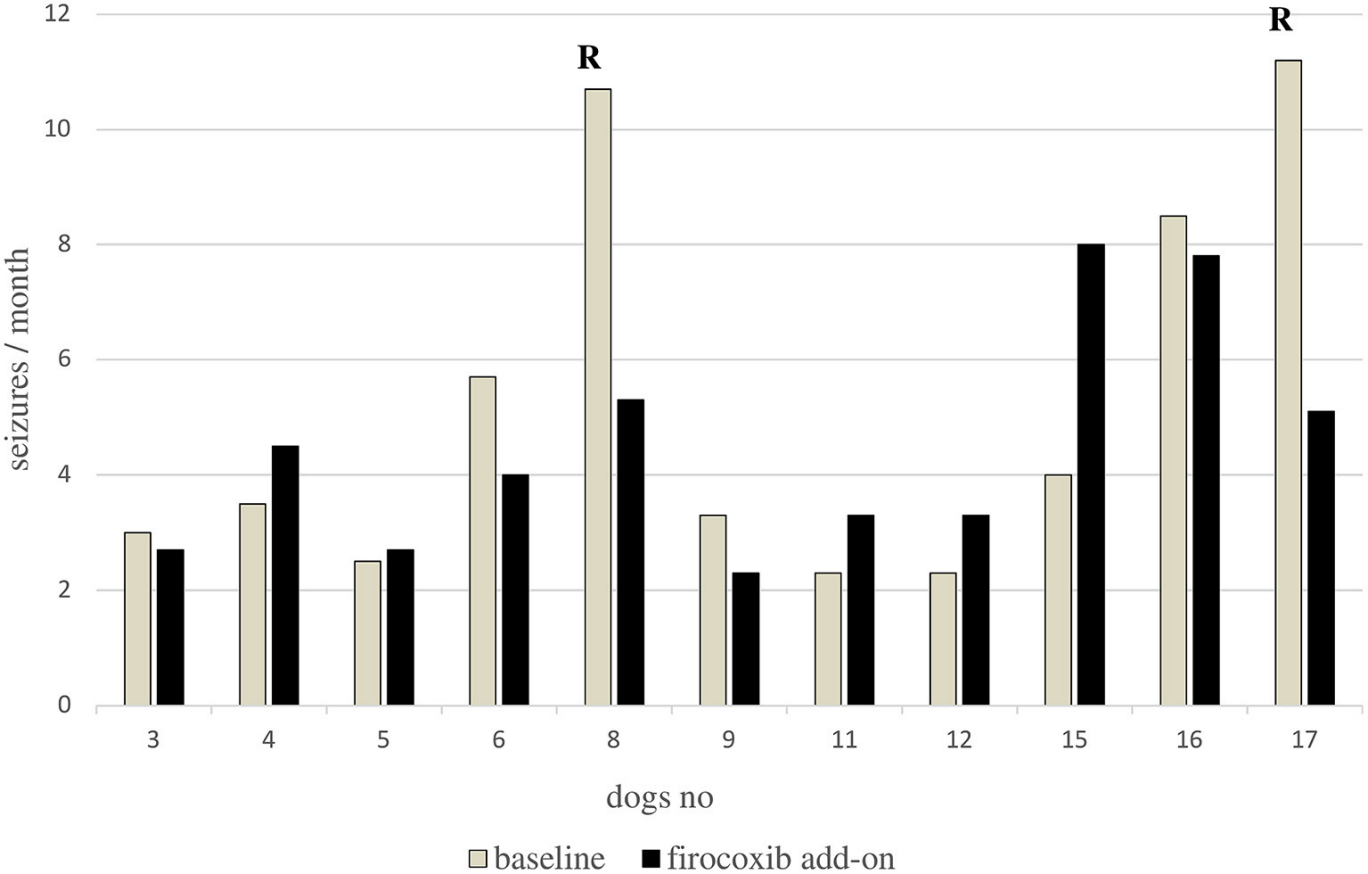
Bar graph showing the individual monthly seizure frequency at baseline and during firocoxib add-on treatment. Six dogs showed a decrease in seizure frequency and five dogs showed an increase in seizure frequency. Only two dogs (cases 008,017) were classified as responders (R) defined by a seizure frequency reduction of ≥50%. None of the dogs became seizure-free.

The seizure frequency of the study population with pooled data (*n* = 11) was not relevantly altered by the firocoxib add-on treatment strategy [median seizure frequency (range): baseline 3.5 seizures/month (2.3–11.2); firocoxib add-on 4 seizures/month (2.3–8); *p* = 0.59] during the six-month treatment period (Figure 2A). However, considering individual data, six dogs (55%, 6/11) experienced a seizure frequency reduction in response to firocoxib add-on therapy ranging from 8 to 54% (Figure 3). Among these, two dogs (dogs #8, #17) showed a seizure frequency reduction of ≥ 50%. These were the dogs that suffered from the highest baseline seizure frequency (10.7 and 11.2 seizures/month, respectively), indicating that Pgp-mediated mechanisms may be only clinically relevant in a proportion of phenobarbital-resistant dogs with a very high baseline seizure frequency.

Considering that the frequent occurrence of cluster seizures and a high seizure density seems to be linked to a poor drug responsiveness in canine epilepsy patients and upregulation of p-glycoprotein and that cluster seizures have a major impact on quality-of-life in epilepsy patients, we separately assessed an effect on occurrence of cluster seizures defined as days with cluster seizures. There was no relevant alteration in cluster frequency with respect to the pooled data of the study population during firocoxib add-on treatment [*n* = 11; median cluster frequency (range): baseline 0.8 cluster seizure days/month (0–2); firocoxib add-on 1.0 cluster seizure days/month (0–2); *p* = 0.5] (Figure 2B). There was also no relevant change in seizures/cluster day (median 2.5 seizures/cluster day during baseline vs. 2.6 seizures/cluster day during firocoxib add-on treatment). Considering individual data, the number of days with cluster seizures increased in six dogs during the firocoxib add-on treatment and decreased in four dogs. One dog did not exhibit cluster seizures during the complete study phase. Yet again, one of the dogs with the highest baseline cluster day frequency (#17) showed a reduction of ≥50% in monthly cluster days.

The regular clinical and laboratory controls revealed no relevant or adverse effects of the firocoxib add-on treatment. Two of the dogs developed diarrhea, which was likely related to other reasons (1 endoparasites, 1 infectious gastroenteritis because other dogs in the household were also affected). Intermittent normoglycemic glucosuria was observed in one dog. Mean phenobarbital serum concentration was 29.05 μg/ml prior to study entry and 31.74 μg/ml after 4 months of treatment (10 dogs). However, fluctuations were observed, and trough samples were not consistently obtained.

## Discussion

This pilot study in dogs with phenobarbital-resistant idiopathic epilepsy does not confirm COX-2 targeting with firocoxib as a potent add-on therapeutic strategy for drug-resistant epilepsy. As mentioned above earlier findings from several rodent studies pointed to beneficial effects of COX-2 inhibition with an influence on seizure duration or seizure thresholds (2–4). Moreover, transient COX-2 inhibitor treatment significantly improved the response to phenobarbital in a chronic rodent model of drug-resistant epilepsy suggesting beneficial effects of COX-2 inhibition (5). However, another experimental study with administration of the COX-inhibitor SC-58236 pointed to putative detrimental effects of COX-2 inhibition in the chronic phase of a poststatus epilepticus model with recurrent spontaneous seizures (17). In line with this finding, COX-2 inhibition aggravated seizure activity in a kainate-induced status epilepticus model (18–20).

The differences in the outcome between the experimental studies and the present findings in the dog model might be related to several factors including differences between species and strains as well as in the etiology, seizure history, and intrinsic disease severity. Especially, the disease duration (in our study mean disease duration was 2.1 years) might play an important role in the evaluation of new therapeutic strategies, as there might be a “point of no return” regarding certain epileptogenic pathways in the chronic epileptic brain.

Interestingly, the two dogs with partial treatment success that showed a relevant ≥50% reduction in seizure frequency during firocoxib treatment, exhibited the highest baseline seizure frequency. These data might indicate that only patients with a high intrinsic severity and high seizure density can be benefitted from COX-2 inhibitor as an additional add-on treatment strategy. However, considering the small number of dogs in this pilot study, the natural fluctuation of seizure frequency, and the potential for pseudo responders if some seizures are not documented, controlled studies in larger cohorts are required to confirm a link between seizure frequency and efficacy (21).

Provided that this finding receives further confirmation, it would be in line with the concept that the control of Pgp expression rates might be one factor contributing to the COX-2 inhibitor effects in the epileptic brain (22). Seizures are known to cause only a transient increase of Pgp, which in the rodent models might only last 1–2 weeks following a seizure (23). Thus, accumulation of Pgp induction resulting in a functionally relevant overexpression might only occur in the patients with a high seizure density. This is in line with observations from our pilot data where two dogs with a very high baseline seizure frequency responded with a >50% seizure frequency reduction. Interestingly, phenobarbital concentrations revealed no relevant change in the two dogs with partial treatment success, while we observed an overall increase in phenobarbital during the 6 months treatment period. However, completely different disease-associated factors linked with the intrinsic severity might also contribute to an effect of COX-2 inhibition or to the modulation of seizure activity in these two dogs (24).

In conclusion, our data argue against an overall relevant efficacy of COX-2 inhibitor add-on approaches in the canine phenobarbital-resistant. Overall data may only support a transitory beneficial effect in dogs with very high seizure frequencies which necessitates further confirmation in the controlled studies. Further evaluation and development of respective approaches may be considered in dogs with a very high seizure density.

## Data Availability Statement

The original contributions presented in the study are included in the article, further inquiries can be directed to the corresponding author.

## Ethics Statement

The animal study was reviewed and approved by the hospital board of the Clinics of Small Animal Medicine, Ludwig-Maximilians-University Munich, Germany and the Ethics Committee of the Royal Veterinary College, London, UK. Written informed consent was obtained from the owners for the participation of their animals in this study.

## Author Contributions

HP, HV, and AF designed and supervised the study. VH and VM supervised case collections. AT, MK, FK, FG, and LA contributed cases. HP and VH wrote the first draft of the manuscript. AF finalized the manuscript. All authors provided input into the final version of the manuscript. All authors contributed to the article and approved the submitted version.

## Funding

This study was supported by a grant from the Gesellschaft zur Förderung kynologischer Forschung e V and a Bavarian equal opportunities sponsorship (VH) and was part of a thesis (VM) (25). Research in Heidrun Potschka's group focusing on the neuroinflammation in experimental epilepsy models and in canine epilepsy is supported by Deutsche Forschungsgemeinschaft (PO681/8-1).

## Conflict of Interest

The authors declare that the research was conducted in the absence of any commercial or financial relationships that could be construed as a potential conflict of interest.

## Publisher's Note

All claims expressed in this article are solely those of the authors and do not necessarily represent those of their affiliated organizations, or those of the publisher, the editors and the reviewers. Any product that may be evaluated in this article, or claim that may be made by its manufacturer, is not guaranteed or endorsed by the publisher.

## Footnotes

^1^Previcox^®^ (Boehringer Ingelheim) licensed for the control of pain and inflammation associated with osteoarthritis, soft-tissue, orthopedic, and dental surgery in dogs (EMA/CVMP/19896/2007&EMEA/V/C/000082).
